# The prevalence and role of human papillomavirus genotypes in primary cervical screening in the northeast of China

**DOI:** 10.1186/1471-2407-12-160

**Published:** 2012-05-01

**Authors:** Shizhuo Wang, Heng Wei, Ning Wang, Shulan Zhang, Yao Zhang, Qiang Ruan, Weiguo Jiang, Qian Xiao, Xiaomei Luan, Xiuyan Qian, Lili Zhang, Xiang Gao, Xiaowei Sun

**Affiliations:** 1 Department of Obstetrics and Gynecology, Shengjing Hospital, China Medical University, Shenyang, Liaoning, China; 2 Virus Laboratory, Shengjing Hospital, China Medical University, Shenyang, Liaoning, China; 3 Department of Pathology, Shengjing Hospital, China Medical University, Shenyang, Liaoning, China; 4 Division of Pediatric Neurology, Weill Cornell Medical College, New York, NY, 10065, USA

**Keywords:** Human papillomavirus genotype, Cervical screening, Cervical cancer, Prognosis

## Abstract

**Background:**

Studies have shown that type-specific persistence of high-risk human papillomavirus (HPV) infection contributed significantly to cervical carcinogenesis.

**Methods:**

In this population-based study (on 24041 women), we report on the prevalent genotypes of HPVs and the prevalent genotypes of HPV persistent infection in the northeast of China.

**Results:**

Our results showed that in HPV infected women (45.6% in total), (95% CI, 44.97%–46.23%), 17.35% (95%CI, 16.87%–17.83%) suffered persistent infection. The most common high-risk HPV types in persistent positivity were HPV-16 (18.21%; 95%CI, 17.04%–19.38%), HPV-58 (13.2%; 95%CI, 12.17%–14.23%), HPV-18 (8.66%; 95%CI, 7.81%–9.51%), HPV-52 (7.06%; 95% CI, 6.28%–7.84%) and HPV-33 (6.78%; 95% CI, 6.02%–7.54%). The prevalence of persistent infections with HPV-16,–58, −18, −52 and 33 in cervicitis were lower compared to those in CIN (all P < 0.05). HPV-58, −33 and multiple HPV persistent positivity were significantly associated with older age (all P < 0.05). HPV-18 persistent positivity was significantly associated with adenocarcinoma and lymphatic metastasis (all P < 0.05). HPV-18 persistent positivity was associated with cervical cancer prognosis (P <0.0001). Multivariate analyses showed that HPV-18 persistent positivity, (RR = 1.704, 95%CI = 1.095–2.654, p = 0.028) and lymphatic metastasis (RR = 2.304, 95%CI = 1.354–3.254, P = 0.015) were independent predictors for 3-year survival in cervical cancer.

**Conclusions:**

we provided extensive results of HPV genotype prevalence and distribution in the northeast of China. HPV genotyping is worthwhile to perform because of its independent prognostic value in cervical cancer

## Background

Human Papillomavirus (HPV) infection usually proceeds for the development of virtually all invasive cervical cancers, their associated precancerous lesions, and genital warts [1,2]. To date, more than 200 HPV genotypes have been identified, but the interest is focused only on genital HPVs (40 genotypes) that are associated with precancerous and cancerous lesions of the cervix [3,4]. Although high incidence and prevalence are found in females after the onset of sexual activity, most of HPV infection are transient and clear within 6–12 months. Studies showed that type-specific persistence of high-risk human papillomavirus (HPV) infection contributed significantly to cervical carcinogenesis [5-7].

It is expected that the next-generation approach of HPV vaccines may more efficiently prevent cervical cancer, however, efforts to implement and evaluate a vaccination strategy are dependent on our understanding of the behaviors of HPV type-specific infection. Meanwhile, the geographic variation in the prevalence and distribution of HPV genotypes had been reported in different countries, even different regions in the same country. It’s necessary for us to study the prevalence and role of human papillomavirus genotypes in primary cervical screening in different geographic regions [8,9]. We have previously reported the prevalence of HPVs in 1444 Women in Liaoning Province, China [10]. However, no large epidemiologic data has been reported in the northeast of China.

We performed a population-based study (24041 women) to investigate the prevalent high-risk genotypes of HPVs and the results in HPV infected women in the northeast of China. After they first visited, we randomly selected 15257 from those 24041 women, and followed them up to test their HPV persistence as well as their pathological changes, in order to study the association between the persistent HPV infections and cervical lesions.

## Methods

### Enrolled group

From 2007 to 2010, 24041 women between the ages of 18 and 60 years who were permanent residents in the northeast of China were eligible to participate. All women were composed of healthy Chinese women who had underwent cervical cancer screening in our Health Check Center or at the Department of Obstetrics and Gynecology, Shengjing Afiliated Hospital of China Medical University. These women were not pregnant during their first visit and also had no intention to be pregnant during the first year of follow-up. Moreover, we excluded patients who had a history of hysterectomy, or were being treated with vaginal medicine at that time or will be treated for cervical diseases in 6 months. To obtain specific data about HPV persistent infection or re-infection, 24041 from 26126 eligible women (92%) were successfully followed-up, patients who died for non-cancer related diseases were excluded from this analysis. As for women with HPV DNA positive were followed-up every 4 months during the first year and subsequently twice a year until 31 Dec 2010; as for women with HPV DNA negative were followed-up once a year until 31 Dec 2010. To analyze the correlation between HPV persistent infection and cervical lesions, HPV screening was applied to the 24041 women. Meanwhile, 15257 women were enrolled for cervical biopsy done by professional pathologists [11]. Subjects gave a signed informed consent. The study protocol was approved by institutional ethical and research review boards of the participating institutions in the northeast of China.

### HPV DNA detection

All HPV tests were performed without the knowledge of cytological or histological interpretation. Human papillomavirus DNA was amplified with the L1 consensus HPV primers. Biotin was used for negative control and an internal control set of biotinylated primers simultaneously amplifies a 268-bp fragment of the human A-globin gene was used for positive control in each reaction as previously described [8].

### HPV genotyping

Human papillomavirus genotyping was performed using the HPV GenoArray test kit (HybriBio Ltd), which was used in both DNA amplification and HybriBio’s proprietary flow-through hybridization technique as previously described. HPV Blot contains 21 types of genotypes, including 5 low-risk types (6, 11, 42, 43, and 44), 14 high-risk types (16, 18, 31, 33, 35, 39, 45, 51, 52, 56, 58, 59, 66, and 68), and 2 intermediate-risk types (CP8304 and 53). HPV L1 and an internal control of the human A-globin in each reaction confirm absence of HPV DNA contamination

### Colposcopy and histological diagnosis

15257 eligible women, corresponding to a 63% response rate, were chosen for a colposcopic examination and cervical biopsy at every subsequent visit. Multiple punch biopsy specimens were taken under colposcopy. Lesions in the transformation zone were assessed by 5% acetic acid and iodine solution. If the colposcopy results proved unsatisfactory for a definitive diagnosis, further exploration of the endocervix was systematically carried out.

### Immunohistochemistry analysis for HPV 18-E6 protein

178 cervical cancer biopsies were fixed in formalin buffer and embedded in paraffin. Sections of 5-mm thickness were obtained from the paraffin blocks, placed on siliconized glass, and left at 37°C overnight. The antibody used to label the HPV-18 E6 protein was the polyclonal antibody (Santa), (1/100 dilution, 30-minute incubation at room temperature). Color development was achieved with the chromogen diaminobenzidine. Sections were counterstained with hematoxylin. Staining was scored by estimating the percentage of cells that had cytoplasm staining for DKK4 and multiplying by the assessment of the intensity of the stain on a 1+, 2+, or 3+ scale. The theoretical limits of the scores ranged from 0 (0% of cells staining) to 300 (100% of the cells staining at 3+ intensity), as described above [12]. All of the stained sections were assessed by two independent pathologists who were unaware of the patients’ clinical data.

### Statistical analysis

The data were analyzed using the SPSS version 13.0 statistical package. Their hazard ratio’s (HRs) and 95% CIs were calculated using the Wald test. Pearson’s *χ*^2^ test was used to evaluate the association between covariates. Cumulative the overall survival time was calculated by the Kaplan–Meier method and analyzed by the log-rank test. Multivariate analyses were based on the Cox regression model. All tests were two sided, and P <0 .05 was considered statistically significant.

## Results

### The prevalence and genotypes of human papillomavirus in the northeast of China

In this study, persistent positivity was defined as the detection of the same HPV genotypes over three subsequent visits, whereas transient positivity was defined as the detection of the same high-risk HPV genotypes no more than three visits. A total of 24041 participants were successfully enrolled for HPV screening from 2007 to 2010 as mentioned above. Our results found that 45.6%; (95% CI, 44.97%–46.23%) was positive for HPV DNA. Among those HPV positive women, 17.35%; (95% CI, 16.87%–17.83%) suffered persistent infection and 28.25%; (95% CI, 27.68%–28.82%) suffered transient infection. Furthermore, we studied the prevalent genotypes of HPV persistent positivity. Among the women who were HPV persistent positivity (17.35% of total), 14.16%; (95% CI, 13.78%–14.54%) were found to have a single HPV genotype. On the other hand, two HPV genotypes were 2.77%; (95% CI, 2.97%–3.41%) and 0.34%; (95% CI, 0.27%–0.41%) infected with three HPV genotypes and 0.08%; (95% CI, 0.04%–0.12%) infected with more than three HPV genotypes, 3.19%; (95% CI, 2.97%–3.41%) women were found more than one HPV type at the same or different times;.

**Figure 1:**
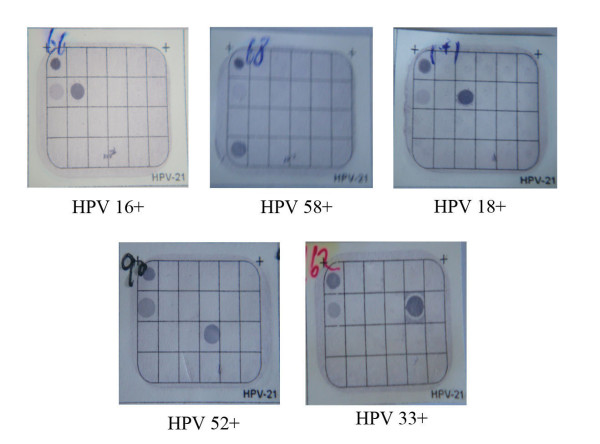
**The common high-risk genotypes of persistent positive HPV in the northeast of China.** Human papillomavirus genotyping was performed using the HPV GenoArray tests. The common high-risk genotypes of HPV in persistent positivity were HPV-16 (18.21%; 95% CI, 17.04%–19.38%), HPV-58 (13.2%; 95% CI, 12.17%–14.23%), HPV-18 (8.66%; 95% CI, 7.81%–9.51%), HPV-52 (7.06%; 95% CI, 6.28%–7.84%) and HPV-33 (6.78%; 95% CI, 6.02%–7.54%).

All of the 21 different HPV genotypes were found in the persistently infected samples. Among these types, the most common high-risk HPV types in persistent positivity were HPV-16 (18.21%; 95% CI, 17.04%–19.38%), HPV-58 (13.2%; 95% CI, 12.17%–14.23%), HPV-18 (8.66%; 95% CI, 7.81%–9.51%), HPV-52 (7.06%; 95% CI, 6.28%–7.84%) and HPV-33 (6.78%; 95% CI, 6.02%–7.54%) (Figure 1). Other high-risk HPV types were found with a frequency of no more than (5%; 95% CI, 0.69%–4.91%).

### The association between prevalent genotypes of human papilloma virus persistent positivity and cervical lesions

In order to analyze the correlation between prevalent genotypes of HPV persistent infection and cervical lesions, 15257 eligible women, corresponding to a 63% response rate, were chosen for cervical biopsy as mentioned above. The results were shown in Table 1.

**Table 1:** The association between prevalent genotypes of human papilloma virus persistent positivity and cervical lesions

																						
**Pathological stage**	**No. 15257**	**HPV +**	***χ***^**2**^	**P**	**Multiple HPV(+)**	***χ***^**2**^	**P**	**HPV 16+**	***χ***^**2**^	**P**	**HPV 58+**	***χ***^**2**^	**P**	**HPV 18+**	***χ***^**2**^	**P**	**HPV 52+**	***χ***^**2**^	**P**	**HPV 33+**	***χ***^**2**^	**P**
**Stage 1**																						
**normal**	**10477**	**262**	**519.846**	**0.0001**	**98**	**404.940**	**0.0001**	**43**	**139.334**	**0.0001**	**59**	**494.956**	**0.0001**	**83**	**45.796**	**0.0001**	**55**	**59.537**	**0.0001**	**47**	**94.149**	**0.0001**
**CINI**	**2068**	**283**			**162**			**62**			**155**			**51**			**45**			**52**		
**Stage 2**																						
**CINI**	**2068**	**283**	**2054.183**	**0.0001**	**162**	**107.486**	**0.0001**	**62**	**180.252**	**0.0001**	**155**	**0.444**	**0.522**	**51**	**5.929**	**0.016**	**45**	**4.881**	**0.031**	**52**	**3.019**	**0.094**
**CINII/III**	**1604**	**1426**			**311**			**247**			**111**			**62**			**54**			**56**		
**Stage 3**																						
**CINII/III**	**1604**	**1426**	**0.53**	**0.5**	**311**	**1.245**	**0.271**	**247**	**0.046**	**0.829**	**111**	**0.321**	**0.571**	**62**	**6.374**	**0.013**	**54**	**1.999**	**0.157**	**56**	**0.677**	**0.378**
**Cervical cancer**	**1108**	**975**			**196**			**174**			**83**			**66**			**49**			**42**		

Among 15257 cervical specimens, the pathologic diagnoses were made as follows: 10477 as cervicitis, 2068 were CIN I, 1604 were CIN II/III and 1108 were cervical cancer (CC). Our results showed that, the prevalence of HPV persistent infection in cervicitis, CIN I, CIN II/III, and CC was 2.5%; (95% CI, 2.2%–2.8%), 13.68%; (95% CI, 12.2%–15.16%), 88.9%; (95% CI, 87.36%–90.44%) and 88%; (95% CI, 86.09%–89.91%) respectively. The prevalence of HPV in persistent infection in cervicitis was significantly lower than that in CIN I. The HPV prevalence in CINI was significantly lower than that in CIN II/III. However, there was no significant difference between HPV persistent infection in CIN II/III and that in CC. The prevalence of common HPV types in persistent infection was HPV-16,-58,-18,-52,-33 (as mentioned above). In the women with HPV-16 infection, the prevalence in cervicitis (0.41%; 95%CI, 0.29%–0.53%) was lower than that in CIN I (3%; 95% CI, 2.26%–3.74%), (P < 0.0001). HPV-16 in CIN I was significantly lower than that in CIN II/III (15.4%; 95% CI, 13.63%–17.17%), (P < 0.0001). Same as in the multi-high-risk HPV infection, there was no significant difference between HPV-16 persistent infection in CIN II/III and that in CC (15.7%; 95% CI, 13.56%–17.84%), (P = 0.829). However, there was some difference in HPV-58 and HPV-33 infection. Although it was significantly lower in cervicitis with HPV-58 (0.56%; 95% CI, 0.42%–0.70%) and HPV-33 (0.45%; 95% CI, 0.32%–0.58%) compared to in CIN I infected with HPV-58 (7.50%; 95% CI, 6.36%–8.64%), (P < 0.0001) and HPV-33 (2.51%; 95% CI, 1.84%–3.18%), (P < 0.0001), there were no significant differences when comparing between in CINI and in CIN II/III with infection of HPV-58 (6.92%; 95% CI, 5.68%–8.16%), (P = 0.522), as well as HPV-33 (3.49%; 95% CI, 2.59%–4.39%), (P = 0.094). It was similar in the groups of CIN II/III and CC. There were no significant differences in HPV-58 (7.49%; 95% CI, 5.94%–9.04%), (P = 0.571) versus in HPV-33 (3.79%; 95% CI, 2.91%–4.91%), (P = 0.378). HPV-18, -52, and multiple HPV infection had alike patterns. In details, HPV-18 persistent positivity in cervicitis (19.39%; 95% CI, 17.46%–21.32%) was significantly lower as compared to that in CIN I (2.47%; 95% CI, 1.8%–3.14%), (P < 0.0001), HPV-52 persistent positivity in cervicitis (0.52%; 95% CI, 0.38%–0.66%) was significantly lower as compared to that in CIN I (2.23%; 95% CI, 1.65%–2.95%), (P < 0.0001), and multiple HPVs persistent positivity in cervicitis (0.94%; 95%CI, 0.76%–1.12%) was also significantly lower as compared to that in CIN I (7.38%; 95% CI, 6.25%–8.51%), (P < 0.0001). The prevalence of HPV-18 in CINI was significantly lower than that in CIN II/III (3.86%; 95% CI, 2.92%–4.8%), (P = 0.016), HPV-52 in CINI was significantly lower than that in CIN II/III (3.37%; 95% CI, 2.12%–4.62%), (P = 0.031) and multiple HPVs in CINI was also significantly lower than that in CIN II/III (19.39%; 95% CI, 17.46%–21.32%), (P < 0.0001) . Exceptionally, the prevalence of HPV-18 persistent infection in CIN II/III was significantly lower than that in CC (6.33%; 95% CI, 1.43%–7.76%), (P = 0.013). However, there was no significant difference between HPV-52 persistent infection in CIN II/III and that in CC (4.42%; 95% CI, 1.21%–5.63%), (P = 0.157) as well as multiple HPV infection in CIN II/III to in CC (21.49%; 95% CI, 19.07%–23.91%), (P = 0.271).

### The association between prevalent genotypes of human papilloma virus persistent positivity and cervical cancer prognosis

Among 15257 eligible women chosen for cervical biopsy, 1108 women were diagnosed to have CC. We studied the associations of HPV persistent infection with various clinicopathologic characteristics (including age, FIGO stage, Grade, lymph metastasis, histology type). The results were shown in Table 2.

**Table 2:** The association between prevalent genotypes of human papilloma virus persistent positivity and cervical cancer clinical pathological data

																						
**Data**	**No.** **1108**	**HPV** **+**	***χ***^**2**^	**P**	**Multiple** **HPV(+)**	***χ***^**2**^	**P**	**HPV** **16+**	***χ***^**2**^	**P**	**HPV** **58+**	***χ***^**2**^	**P**	**HPV** **18+**	***χ***^**2**^	**P**	**HPV** **52+**	***χ***^**2**^	**P**	**HPV** **33+**	***χ***^**2**^	**P**
**Age**																						
**<35**	**376**	**326**	**0.903**	**0.380**	**53**	**5.048**	**0.025**	**69**	**3.013**	**0.097**	**18**	**6.004**	**0.016**	**21**	**0.140**	**0.789**	**14**	**0.658**	**0.446**	**6**	**7.518**	**0.005**
**≥35**	**732**	**649**			**143**			**105**			**65**			**45**			**35**			**36**		
**FIGO stage**																						
**Ia~Ib**	**643**	**562**	**0.511**	**0.513**	**115**	**0.040**	**0.873**	**91**	**2.786**	**0.112**	**55**	**2.497**	**0.133**	**38**	**0.006**	**1.000**	**28**	**0.017**	**0.884**	**26**	**0.269**	**0.636**
**IIa~IV**	**465**	**413**			**81**			**83**			**28**			**28**			**21**			**16**		
**Grade**	**1069**																					
**G1 ~ G2**	**531**	**479**	**1.314**	**0.28**	**109**	**3.387**	**0.069**	**81**	**0.810**	**0.407**	**40**	**0.003**	**1.000**	**30**	**0.501**	**0.526**	**25**	**0.037**	**0.884**	**22**	**0.472**	**0.523**
**G3**	**538**	**496**			**87**			**93**			**41**			**36**			**24**			**18**		
**Lymph metastasis**	**1043**																					
**no**	**739**	**692**	**0.800**	**0.417**	**135**	**0.094**	**0.792**	**113**	**3.533**	**0.068**	**54**	**1.077**	**0.312**	**36**	**9.074**	**0.005**	**34**	**0.000**	**1.000**	**27**	**0.914**	**0.386**
**yes**	**304**	**280**			**58**			**61**			**28**			**30**			**14**			**15**		
**Histology**																						
**Squamous** **carcinoma**	**767**	**681**	**1.477**	**0.230**	**129**	**1.298**	**0.268**	**121**	**0.010**	**1.000**	**57**	**0.013**	**0.902**	**38**	**4.469**	**0.039**	**35**	**0.117**	**0.874**	**28**	**0.134**	**0.734**
**Adeno-** **carcinoma**	**341**	**294**			**67**			**53**			**26**			**28**			**14**			**14**		

**Figure 2:**
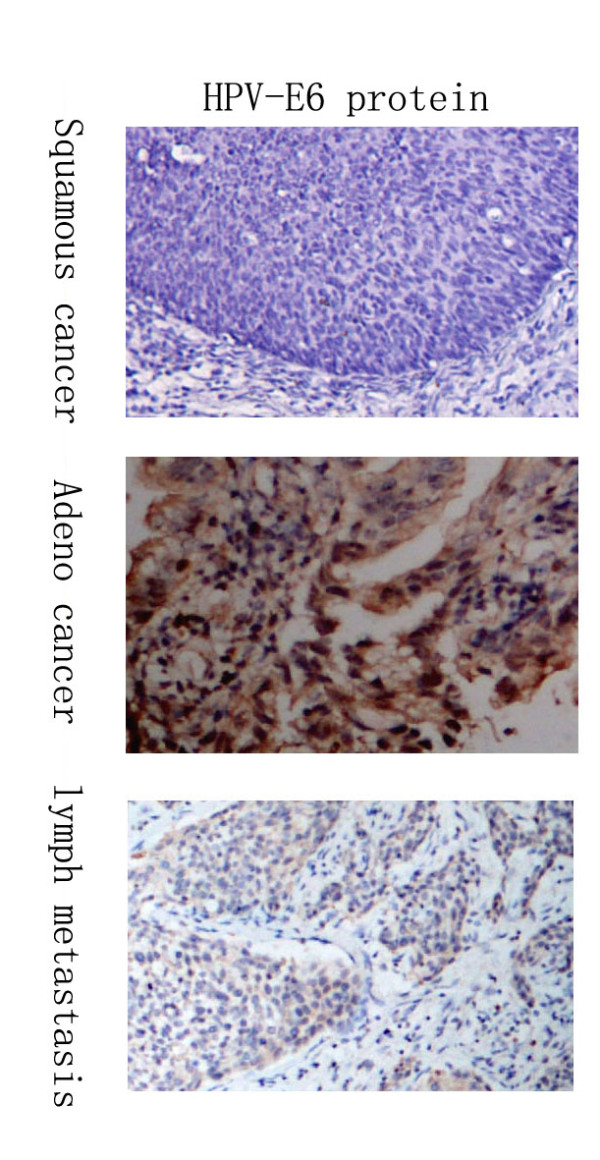
**Immunohistochemistry analysis showed the expression of HPV-E6 protein in 178 cervical cancer samples**. HPV-E6 protein expression in 79 squamous samples was significantly lower as compared with those in 99 adeno samples. (*χ*2 = 4.214, P = 0.04). HPV-E6 protein was correlated with lymph metastasis. (*χ*2 = 16.049, P < 0.001).

Our results found that there were no significant differences between HPV persistent positivity and clinicopathologic characteristics (all P > 0.05). The associations between the genotypes of HPV persistent positivity and various clinicopathologic characteristics were also studied. The results showed that there were no significant differences between HPV-16, -52 persistent positivity and clinicopathologic characteristics (all P > 0.05). HPV-58, -33 and multiple HPV persistent positivity were significantly associated with older age (all P < 0.05). HPV-18 persistent positivity was significantly associated with adenocarcinoma and lymphatic metastasis (all P < 0.05). Meanwhile, immunohistochemical staining found that HPV-E6 protein was positively correlated with adenocarcinoma and lymphatic metastasis (P < 0.001) (Figure 2). While not associated with age, differentiation, or FIGO stage (all P > 0.05) (Table 3).

**Table 3:** Relationships between HPV-E6 and clinicopathological facotrs in 178 cases of cervical cancers

	**No.**	**HPV-E6 protein expression**			
		**++**	**-,+**	***χ***^**2**^	**P**
**Age**					
**<45**	**58**	**40**	**18**	**0.546**	**0.505**
**≥45**	**120**	**76**	**44**		
**Histology**					
**Squamous carcinoma**	**79**	**45**	**34**	**4.214**	**0.04**
**adenocarcinoma**	**99**	**71**	**28**		
**Differentiation**					
**G1 ~ G2**	**89**	**56**	**33**	**0.396**	**0.529**
**G3**	**89**	**60**	**29**		
**Lymph metastasis**					
**yes**	**67**	**56**	**11**	**16.049**	**0.001**
**no**	**111**	**60**	**51**		
**FIGOstage**					
**I~IIa**	**129**	**82**	**47**	**0.530**	**0.467**
**IIb~IV**	**49**	**34**	**15**		

**Figure 3:**
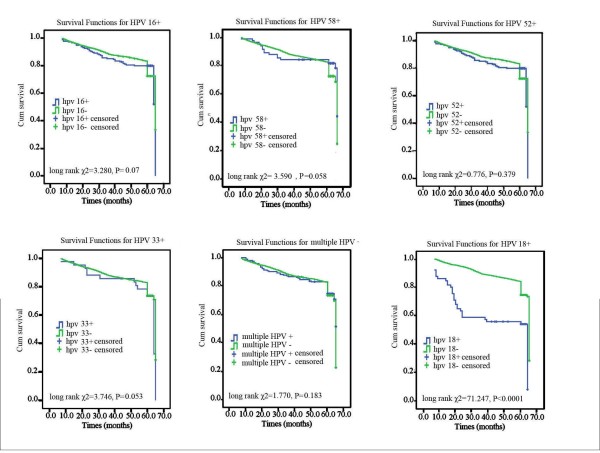
**The association between prevalent genotypes of human papilloma virus persistent positivity and cervical cancer prognosis.** HPV-16, -58, -52, -33 or multiple HPV persistent positivity were not associated with the survival times of CC patients (all P > 0.05). (all P > 0.05). However, the mean ± SD of the survival time for CC patients with HPV-18 persistent positivity (43.294 ± 2.978months) was significantly lower than that for CC patients with HPV-18 transient positivity or negativity (59.271 ± 0.415months), with P < 0.0001.

Meanwhile, we also studied the association between type-specific HPV persistent infection and the overall 3-year survival times for CC patients. The results of the overall 3-year survival time for type-specific HPV persistent infection were shown in Figure 3. HPV-16, -58, -52, -33 or multiple HPV persistent positivity were not associated with the overall 3-year survival times for CC patient (all P > 0.05). The mean ± SD of the overall 3-year survival time for CC patients with HPV-18 persistent positivity (43.294 ± 2.978months) was significantly lower than that for CC patients with HPV-18 transient positivity or negativity (59.271 ± 0.415months), with P < 0.0001. HPV-18, lymphatic metastasis, pathological grade, FIGO stage and age were chosen for Cox regression. Multivariate analyses showed that the HPV-18, (OR = 1.704, CI = 1.095–2.654, p = 0.028) and lymphatic metastasis (OR = 2.304, CI = 1.354–3.254, P = 0.015) were independent predictors for 3-year overall survival time.

Meanwhile, we also studied the association between type-specific HPV persistent infection and the overall 3-year survival times for CC patients. The results of the overall 3-year survival time for type-specific HPV persistent infection were shown in Figure 3. HPV-16, -58, -52, -33 or multiple HPV persistent positivity were not associated with the overall 3-year survival times for CC patient (all P > 0.05). The mean ± SD of the overall 3-year survival time for CC patients with HPV-18 persistent positivity (43.294 ± 2.978months) was significantly lower than that for CC patients with HPV-18 transient positivity or negativity (59.271 ± 0.415months), with P 0.0001. HPV-18, lymphatic metastasis, pathological grade, FIGO stage and age were chosen for Cox regression. Multivariate analyses showed that the HPV-18, (OR = 1.704, CI = 1.095–2.654, p = 0.028) and lymphatic metastasis (OR = 2.304, CI = 1.354–3.254, P = 0.015) were independent predictors for 3-year overall survival time.

## Discussion

Cervical cancer remains the second most common malignancy in women in the world [13]. Persistent HPV infections cause virtually all of the more than 500,000 cases of invasive cervical cancer per year worldwide [14]. The natural history of HPV is the basis for the rational use of preventive measures [15,16]. Until now, no large epidemiologic data on the prevalence and role of human papillomavirus genotypes in cervical screening had been reported in the northeast of China. In this project, we applied a population-based study (26126 women) for reporting the prevalent genotypes of HPV and the prevalent HPV persistent infection in the northeast of China.

Our results showed that in HPV infected women (45.6% in total), (95% CI, 44.97%–46.23%), 17.35% (95% CI, 16.87%–17.83%) suffered persistent infection. A total of 21 HPV genotypes were detectable in this study, including 14 high-risk types (16, 18, 31, 33, 35, 39, 45, 51, 52, 56, 58, 59, 66, and 68), 2 intermediate-risk types (CP8304 and 53), and 5 low-risk types (6, 11, 42, 43, and 44). Among of these types, the most common HPV types in persistent positivity were HPV-16, HPV-58, HPV-18, HPV-52 and HPV-33.

Furthermore, our results also showed that the prevalence of HPV in persistent infection was increased with the severity of CIN. However, there were no significant differences between HPV persistent infection in CIN II/III and that in CC. The prevalence of HPV-16,-58 and multiple HPV persistent infections in normal cervicitis were significantly lower than those in CIN. There were no significant differences between HPV-16,-58 and multiple HPV persistent infection in CIN II/III and that in CC. Then we hypothesis that HPV-16,-58 and multiple HPV persistent infection may be involved in cervical intraepithelial neoplasia while have little contribution to the carcinogenesis from CIN to cervical cancer. The prevalence of HPV-18 persistent infection was increased with the severity of cervical lesion. The prevalence of HPV-52, -33 persistent infections in normal cervicitis were significantly lower than those in CINI. However, there were no significant differences between HPV-52,-33 persistent infections in CINI and that in CIN II/III and that in CC.

Additionally, HPV-58, -33 and multiple HPV persistent positivity were significantly associated with older age. Recently, some studies have confirmed our results [17,18]. Some considered that older women with different new sexual partners might be tended to infect with multiple HPV persistent positivity [18]. The reason for HPV-58, -33 associated with older age in CC patients was unclear. More studies are also needed to confirm it and clarify the mechanism. HPV-18 persistent positivity was significantly associated with adenocarcinoma and lymphatic metastasis. Meanwhile, the mean ± SD of the survival time for CC patients with HPV-18 persistent positivity was significantly lower than that for patients with HPV-18 transient positivity or negativity. Multivariate analyses showed that the HPV-18 was an independent predictor for 3-year survival. Some studies have reported HPV 18 was significantly associated with clinicopathologic characteristics and prognosis of cervical cancer [19-21]. We recognize that there are several limitations in this study, one of which is the absence of behavioral information. Some reports found that behavioral information in Chinese population play detrimental roles in the development of HPV infection and cervical cancer. Such as, sexual activity, smoking, a diet rich in salty meat, a diet poor in fresh fruit and vegetables or other dietary habits [12,18,22]. And a population-based study about the correlation between behavioral information with cervical cancer will be programmed in our further research. More studies are also needed to confirm it and clarify the mechanism.

## Conclusion

In conclusion, we provided extensive results on HPV genotype prevalence and distribution in the northeast of China. HPV genotyping is worthwhile to perform because of its independent prognostic value in cervical cancer, and the predicting models for death could be useful for counseling the individual patient and stratifying study patients in HPV type-specific vaccines.

## Competing interest

The authors declare they have no competing interests.

## Authors’ contribution

SW carried out the HPV DNA detection, participated in the sequence alignment and drafted the manuscript. HW carried out the HPV DNA detection,and participated in collected cases. NW carried out the immunohistochemistry analysis,and participated in collected cases. SZ participated in the sequence alignment and drafted the manuscript,statistical analysis and experimental guidance. YZ carried out the colposcopic examination. QR carried out the HPV DNA detection and human papillomavirus genotyping analysis. WJ carried out the immunohistochemistry analysis. QX participated in collected cases. XL participated in collected cases. XQ participated in collected cases. LZ participated in collected cases. XG participated in collected cases. XS participated in collected cases. All authors read and approved the final manuscript.

## Pre-publication history

The pre-publication history for this paper can be accessed here:

http://www.biomedcentral.com/1471-2407/12/160/prepub
